# Organisation and provision of head and neck cancer surgical services in the United Kingdom: United Kingdom National Multidisciplinary Guidelines

**DOI:** 10.1017/S0022215116000839

**Published:** 2016-05

**Authors:** F Stafford, K Ah-See, M Fardy, K Fell

**Affiliations:** 1 Sunderland Royal Hospital, Sunderland, UK; 2 Department of Otolaryngology – Head and Neck Surgery, Aberdeen Royal Infirmary, Aberdeen, UK; 3 University Hospital of Wales, Cardiff, UK; 4 NHS England (Midlands and East), CNS Tumours and Head and Neck Cancers, UK

## Abstract

This is the official guideline endorsed by the surgical specialty associations involved in the care of head and neck cancer patients in the UK. This paper summarises the current state of play in the organisation and provision of head and neck cancer surgical services in the UK.

## Introduction

The quality and availability of care for patients with head and neck cancer has improved immeasurably over the past 30 years. Improved training, application of evidence-based practice, multi-disciplinary working, improved surgical and radiation techniques, chemotherapy, public health education, subspecialisation and in particular the National Institute for Health and Care Excellence (NICE) Improving Outcomes guidelines,^1^ the previous editions of the Multidisciplinary Head and Neck Cancer guidelines^2^ and peer review have all played their part. Despite this, the availability of some treatment options and survival outcomes in the UK still seem to lag behind other Western countries. Further improvement is required but the financial constraints in the National Health Service (NHS), highlighted over recent months, could overwhelm us and consequently could affect progress in developing clinical services for the foreseeable future.

Since the inception of the NHS, healthcare spending in the UK has increased 4 per cent per year. In 1960, it was less than 5 per cent of gross domestic product (GDP), 50 years on it is now about 10 per cent of GDP. Current estimates suggest that within 10 years, unchecked healthcare spending will outstrip economic growth and is not sustainable, and by 2050 spending would increase to over 20 per cent of GDP.^3^ The Five Year Forward View, published in October 2014,^4^ describes ways in which the NHS intends to tackle the exponential rises in the cost of NHS services.

## Commissioning healthcare services

### England

Commissioning of healthcare in all its aspects underwent a total organisational restructuring based upon recommendations in the Health and Social Care Act 2012.^5^ This is the fifth major reorganisation of the NHS structure since 2000. Primary Care Trusts and Strategic Health Authorities were disbanded and replaced with 211 Clinical Commissioning Groups (CCGs) made up of local GPs covering populations of over 250 000 under the umbrella of The NHS Commissioning Board, which became NHS England and began functioning on 1st April 2013. Clinical Commissioning Groups do not commission GP or specialised services as these are directly commissioned.^6^ Some services have been designated as ‘specialised’ and based upon principles laid out in the Carter Report and the Department of Health white paper ‘Equity and excellence: liberating the NHS’.^7^ In addition, a structure for prescribing and identifying these services is now in place.

NHS England became responsible for directly commissioned services (including specialised services) in April 2013 (Scotland and Wales have their own commissioning structures). This structure is currently under review and many of the designated specialised services may have commissioning devolved to the CCGs. The NHS England website defines specialised services as those provided in relatively few hospitals, accessed by comparatively small numbers of patients but with catchment populations of usually more than 1 million. These services tend to be located in specialised hospital trusts that can recruit a team of staff with the appropriate expertise and enable them to develop their skills. Specialised services account for approximately 14 per cent of the total NHS budget, about £13.8 billion per annum. The commissioning of specialised services is a prescribed direct commissioning responsibility of NHS England. The manual for prescribed specialised services 2013/2014 identifies 143 services.^8^

A description of the new structure for commissioning specialised services is given in detail on the NHS England website. Commissioning has been devolved to six programmes of care (POC) each with its own team of commissioners:

• internal medicine
• cancer
• blood and infection
• mental health
• trauma
• women and children.

The national Cancer and Blood POC covers the prescribed specialised services in infection, cancer, immunity and haematology. This relates to both specialised and highly specialised prescribed services, and includes both surgical and medical services. There are 74 specialist services within the POC, and these are clustered into Clinical Reference Groups (CRGs) to support the national work in these areas. The Cancer Programme of Care covers some of the prescribed specialised and highly specialised services. Complex head and neck is one of 17 specialised services in the Cancer and Blood Programme. These service-specific CRGs also work with other CRGs where key service interfaces and interdependencies between CRG areas occur. A public consultation to amalgamate CRGs is currently underway; the impact for head and neck surgery will be the creation of a super CRG that includes all of cancer surgery.

The CRG for a specific specialty will advise the designated commissioners on service standards and requirements, and will complete designated tasks requested by the commissioners. Each CRG consists of a chair and members (up to 15) consisting of representatives from the 12 Clinical Senates, relevant professional organisations and patient groups. England has been divided into 12 Clinical Senates similar (but not identical) geographically to the new Cancer Networks providing members for the different CRGs. More information is available on the NHS England website (http://www.england.nhs.uk/ourwork/part-rel/cs/)

The Complex Head and Neck Clinical Reference Group (HNCRG) covers complex benign and malignant head and neck services and refers to a group of very different tumours, including oral (mouth, lip and oral cavity), larynx, pharynx, thyroid and salivary glands tumours amongst others. It may become the responsibility of the CRGs to advise specialised commissioners and NHS England on ways to improve efficiency and reduce costs without affecting quality or provision of care. An example is the NHS England policy on Transoral Robotic Surgery that has been developed under the aegis of the CRG which is undergoing public consultation at the time of this paper going to press. The apparent poor comparisons with other European cancer outcome audits and a wide national variation in provision of services and outcomes have become powerful drivers for political intervention and change. Thus, over the past 10 years many providers in England have moved towards some forms of centralisation model in response to the National Institute for Health and Care Excellence (NICE) improving outcomes guidance (IOG) for head and neck cancers, although this is not universal.

Potentially, the HNCRG can have a great deal of influence on the future structure of services nationally by setting clear standards to the commissioners who control the funding. To influence this process, readers to contact their respective senate representative. More information about CRGs is available from the NHS England website. http://www.england.nhs.uk/ourwork/commissioning/spec-services/npc-crg/

### Scotland

The NHS in Scotland is a devolved service run by the Scottish government out of parliament in Edinburgh. It is delivered by 14 Regional Health Boards that cover the disparate geography of Scotland. The Scottish NHS budget is approximately £11.9 billion (2013–2014 budget).

Head and neck cancer services are delivered by the three major Cancer Networks within Scotland: North of Scotland cancer network (NOSCAN), South of Scotland (SCAN) and West of Scotland (WOSCAN).

These cancer networks work closely together to provide a full and comprehensive head and neck cancer service to the estimated 5.5 million population in Scotland which is spread across a wide range of geographic areas from dense urban to remote and rural sites. Over 1100 new cases of head and neck cancer are diagnosed in Scotland per year.

In Scotland, commissioning groups have not been introduced in the same way as in England and the delivery of the NHS in Scotland still follows the traditional NHS method of GP referral to the local secondary care centre with ‘urgent suspicion of cancer’ referral guidelines published by NHS Scotland in place. This sets the standard of 62 days from referral to treatment for cancer cases (http://www.healthcareimprovementscotland.org/our_work/cancer_care_improvement/programme_resources/scottish_referral_guidelines.aspx).

Quality improvement processes are in place in Scotland including the introduction of quality performance indicators (QPIs) to set the standards for cancer care within all cancer groups including head and neck cancer. The QPIs have been developed collaboratively with the three Regional Cancer Networks (NOSCAN, SCAN, WOSCAN), Information Services Division and Healthcare Improvement Scotland. The Scottish Government has asked Healthcare Improvement Scotland to provide performance assurance against cancer QPIs and to publish their findings on a three yearly basis (http://www.healthcareimprovementscotland.org/our_work/cancer_care_improvement/cancer_qpis.aspx).

The NHS funding constraints may make it necessary to review the role of the currently designated cancer centres in Scotland in the future.

### Wales

The NHS England has identified head and neck surgery as a speciality requiring specific funding arrangements. The organisation and provision must be based in centres covering a large population with an adequate workload. Over the past 10 years many providers have moved towards some forms of centralisation model in response to the NICE IOG, although this is not universal.

All seven health boards in Wales offer head and neck cancer services, irrespective of numbers; despite an extensive review in 2009 attempting to rationalise services, this has never happened. The existing regional service provision and local geographical and population factors will of course impact on practical arrangements, but trusts will be expected to justify the service structure with robust data. As yet it is not clear if the CRG recommendations, when they are published, will be accepted in Wales and how they will be enforced. It is clear that there will be no increase in funding and only measures which reduce or stabilise costs are likely to be adopted. Head and neck cancer surgical service providers should review their service provision as and when new guidance is published, but no statutory authority exists to enforce these guidelines.

## Cost of head and neck cancer care

Head and neck cancer is expensive to manage. In the USA, it has been suggested that it is the most expensive cancer to treat and patients rarely return to a productive life, with estimated costs of $96 000–$150 000 for multimodality treatment (surgery, chemotherapy and/or radiotherapy).

The UK head and neck surgical services initially developed within ENT and Oral and Maxillofacial (OMF) departments without the introduction of funding and were bundled in with other routine non-oncologic surgical procedures and paid for through local commissioning. Devolvement of services, centralisation and specialisation mean this model cannot continue. Cost estimates for surgery with reconstruction range in the UK and Europe from £25 000 to £30 000 and it is unclear in many units exactly how much of the true cost is reimbursed by current tariffs based on the health resource group codes. We need to be able to quantify the financial impact of CRG advice regarding changes to clinical practice and in order to do this, more clear and more reliable coding and costing is required to understand the viability of services in the future and to monitor the financial effects of change.

## Caseload and service provision

It is generally accepted, with some evidence, that patients requiring complex surgical and oncological treatments have better outcomes and the service is more efficient when carried out in larger centres with specialist surgeons and oncologists. It has already been shown that there is a huge variation nationally in basic measures such as in-hospital mortality and complications which are unacceptable and a major factor in driving change.

The NICE guidance defines a minimum of 100 new cases per year to be a credible provider. The previous edition of the Multidisciplinary Head and Neck Cancer guidelines suggested a higher number, over 250, to generate enough operative cases to develop and maintain skills, provide a suitable training and research environment and allow a sufficient number of qualified surgeons to provide adequate 24 hour, 7 days a week services.

When the 4th edition of these guidelines were published in 2011,^2^ there were 33 cancer networks, with 69 multidisciplinary teams (MDTs) and 79 hospital providers. The 2012 Data for Head and Neck Oncology audit reported 28 MDTs in England, 2 in Wales with 64 service providers for 8272 new cases. Of these providers, 14 reported fewer than 50 new cases per year and a further 11, fewer than 100 new cases. The other units mostly report 150–180 cases, with six providers reporting more than 200 cases per year. There is some under reporting in these numbers due to failure to identify a provider, but the picture is clear. There are units providing head and neck services with relatively low numbers. Trusts and individual surgeons should be examining the sustainability of such service provision outside larger centres. While geographical and public transport issues exist, the CRG agrees with the NICE recommendations for Head and Neck Centres to serve populations of over a million or more. Within England, the CRG's view is that cancer centres should have a case load of at least 250 cases per year, using a regional hub and spoke structure, with centralised surgery and peripheral clinic and support services. Currently, NHS England, in conjunction with National Cancer Intelligence Network, is undertaking an audit of current cancer service provision in England; thus no recommendations will be forthcoming until the audit and the restructuring of the CRG is complete.

Sir Bruce Keogh announced on 16th November 2014 the findings of a forum on provision of a 24 hour, 7 days a week health service, which will filter through to head and neck services eventually (http://www.nhsiq.nhs.uk/improvement-programmes/acute-care/seven-day-services.aspx) and such reorganisation will help with the planned 7-day health service.

Keys to the successful management of HNC are the specialist nursing, speech and language, dietetics and social support that these patients require. Easy and ready access locally is essential. To counterbalance the move to concentrate specialist surgery (radiotherapy is by its nature centralised already) local provision of centrally guided support units and visiting consultant clinics should mitigate some of the patient concerns about distance from the surgical unit.

## Surgical numbers

Individual surgeon reporting is not a concept that is useful or valid in determining outcomes in head and neck surgical practice. However, better outcome measures are on the way and accurate data collection and publication from providers will be required to justify funding, allow comparison with other centres and highlight problems more quickly. Data collection is still poor and undervalued by many hospital managers trying to trim budgets, but the value of accurate validated data cannot be underestimated and it should not be left to busy clinicians to coordinate or enter data but to properly trained and motivated data managers.

Surgeons’ operative numbers is always a thorny question. The peer review process for thyroid surgery adopted the British Association of Endocrine and Thyroid Surgeons guideline of at least 20 thyroidectomies per year as one of its markers and this is likely to be expanded to some of the more common head and neck procedures; examples include neck dissection, oral cancer resection, laryngectomy and free flap reconstruction. There is no real evidence base to determine how many particular procedures should be recommended, but less than five major procedures per year at a centre is not sustainable and a service review is mandatory. Unusual procedures such as craniofacial resection will be restricted even further to a small number of nationally recognised centres.

## Funding

As part of the Five Year Forward View, the commissioning of specialised services will assess the opportunities for co-commissioning across all specialised services in order to maximise all service elements associated with the patients’ pathway and the provision of services to meet the needs of patients. The national service specifications and other commissioning products will provide a framework through which specialised services can be defined to ensure that services are delivered to national standards.

At the time of writing it is not clear what, if any, changes will occur that could affect the commissioning of complex head and neck cancer surgery. At present the commissioning of these services sits within the remit of specialised commissioning, directly commissioned by NHS England. The remaining services are funded by CCGs.

At the start of the specialised commissioning process in 2013, it was clearly stated that the commissioning of these services would be placed in the hands of specialised commissioners, giving the opportunity to ensure that the service delivered was in accordance with the published service specification to ensure equity of access to high-quality services across the country and to aid the smoothing out variations in outcomes noted in cancer audits. There was also a clear belief that this would also drive a more efficient use of funds by providers, potentially a cost reduction through larger services leading to a critical mass of patients supported by an appropriate and cost-effective infrastructure improving the quality and cost-effectiveness in line with the NICE IOG.

Wholesale restructuring of regional services is rarely achievable without cost. This will not be easy and it seems to have stalled the process. Also, such changes are often unwelcome in larger more sparsely populated geographical areas, although evidence would suggest that it is clinicians and providers who provide the most resistance; patients when questioned more often express a desire to go to the expert centre.

## Summary

Complex head and neck surgery has been commissioned as a specialised service by NHS England. The organisation and provision must be based in centres covering a large population with an adequate workload. Over the past 10 years many providers have moved towards some form of centralisation model in response to the NICE IOG, although this is not universal. This is the driver for the work of the CRG, which intends to undertake an audit of the current head and neck services to explore whether the configuration in place meets national IOG requirements and that there is a consistent picture of delivering good outcomes to patients. The view of the CRG is that more centralisation is required.

The existing regional service provision and local geographical and population factors will of course impact on practical arrangements, but trusts will be expected to justify the service structure with robust data. As yet it is not clear if the CRG recommendations will be accepted and how they will be enforced. It is clear that there will be no increase in funding and only measures which reduce or stabilise costs are likely to be adopted. Head and neck cancer surgical service providers should consider their current service provision and assess and consider the potential impact of changes to future guidelines.
